# Understanding Urogenital Schistosomiasis-Related Bladder Cancer: An Update

**DOI:** 10.3389/fmed.2018.00223

**Published:** 2018-08-10

**Authors:** Kenji Ishida, Michael H. Hsieh

**Affiliations:** ^1^Bladder Immunology Group, Biomedical Research Institute, Rockville, MD, United States; ^2^Department of Urology, The George Washington University, Washington, DC, United States; ^3^Division of Urology, Children's National Medical Center, Washington, DC, United States

**Keywords:** cancer, mouse models, schistosomiasis, schistosomiasis haematobia, bladder cancer, bladder

## Abstract

Infection with *Schistosoma haematobium* leads to urogenital schistosomiasis, which has been correlated with the occurrence of bladder cancer. However, mechanisms responsible for this association have not yet been clearly identified. In this short review, we provide an update, highlighting the most recent studies on schistosome-associated bladder cancer, including those that focus on identifying changes in host biology during *S. haematobium* infection, as well as studies for the identification of potentially pro-carcinogenic parasite molecules, and we offer a discussion on some possible mechanisms driving schistosomal bladder cancer.

## *Schistosoma haematobium* and bladder cancer

As with other members of the *Schistosoma* genus, infection with *S. haematobium* leads to schistosomiasis, a debilitating disease affecting more than 200 million people (1). Treatment relies primarily on one drug, praziquantel, and diagnosis methods include egg identification in stool or urine and serum tests for schistosome antigens. Interestingly, and perhaps worryingly, prevalence estimates based on egg identification may underrepresent the actual prevalence, given that such parasitological tests have high specificity but low sensitivity when compared to serum tests (2).

Unlike the more commonly studied *S. mansoni* and *S. japonicum*, which cause hepatosplenic and intestinal pathology, *S. haematobium* causes bladder pathology. While limited evidence exists for the carcinogenic potential of the other schistosome species, the association between *S. haematobium* infection and bladder cancer has been known and studied for many years (3–6). A study conducted in Egypt including nearly 10,000 patients over the period of 1970 to 2007 found a decrease in the frequency of *S. haematobium* infection concomitant with a decrease in squamous cell carcinoma, increase in urothelial cell carcinoma, and increase in the median age of patients (7). In addition to *S. haematobium* infection, other studies have also indicated tobacco smoking as a major risk factor (8, 9). *S. haematobium* joins two other helminth species, *Opisthorchis viverrini* and *Clonorchis sinensis*, as Group 1 definitive biological carcinogenic agents (5, 10), and other parasites from several genera, including *Echinococcus, Strongyloides, Fasciola, Heterakis, Platynosomum*, and *Trichuris* have also been found to be associated with cancer (11).

Regarding the cost of treatment, a recent cost analysis estimated about 18 million USD for praziquantel administration (0.16 USD per person for approximately 112 million people infected with *S. haematobium*), compared to an annual incrementing cost of 20 million USD for the treatment of schistosomal bladder cancer (an estimated 3-4 patients out of 100,000 people infected with *S. haematobium* develop bladder cancer each year) (12). These findings not only emphasize the importance of treating urinary schistosomiasis but they also inspire questions regarding the mechanisms by which *S. haematobium* contributes to the development of bladder cancer [reviewed in (10, 13, 14)]. Here, we highlight recent studies that examine host-side changes in the context of *S. haematobium* infection, as well as studies on potentially carcinogenic parasite molecules (some key studies shown in Table 1), and we also discuss possible mechanisms and future research directions for schistosomal bladder cancer.

**Table 1:** Recent key papers relevant to schistosomal bladder cancer.

**Topic/Findings**	**References**
COX2 and iNOS associated with schistosomal bladder tumor (biopsy)	(15)
EGFR and TGFα associated with schistosomal bladder tumor (biopsy)	(16)
Mouse bladder wall injection method	(17)
(Transcriptome) Change in transcript level of cancer-related genes following mouse bladder wall injection	(18)
(Genome/epigenetics) Change in global gene methylation following mouse bladder wall injection	(19)
(Proteome) Urine proteome profiles ± infection, ± bladder pathology	(20)
(Microbiome) Urine microbiome profiles ± infection, ± bladder pathology	(21)
CHO cells exposed to total worm antigen: proliferation↑, migration↑, apoptosis↓; can generate tumors when injected into nude mice	(22, 23)
Mutation in the *KRAS* gene in bladder tissue that shows dysplasia following intravesical total worm antigen administration	(24)
Host sex-specific effect of p53 haploinsufficiency on urothelial abnormality following bladder wall injection	(25)
Nitrosamine treatment and mouse bladder wall injection resulting in urothelial abnormality and transcription level differences in *CDH1, VIM, MKI67, TP53*	(26)
Estrogen- and guanine-derived metabolites found in the urine of patients infected with *S. haematobium* and in soluble egg antigen	(27, 28)

## Changes in host biology during *S. haematobium* infection

In response to infection with *S. haematobium*, the host bladder undergoes changes at the gross morphological level, as well as at the molecular level. A major goal in the study of the link between *S. haematobium* infection and bladder cancer involves the identification of genes and proteins perturbed following (and during) parasite infection. As a starting point, clinical studies help to identify correlations and possible methods for diagnosis, and they also direct further experiments in animal and *in vitro* models that can validate the clinical findings, allowing us to delve deeper into the molecular pathways involved in schistosomal bladder cancer. For example, some recent studies have used bladder tumor biopsy samples, with and without schistosome eggs, to identify differential levels of host proteins, such as COX2, iNOS, EGFR, and TGFα, that could serve as unique markers for schistosomal bladder cancer (15, 16, 29, 30). Another study examined T cell protein marker expression for STAT4 (Th1), GATA3 (Th2), FOXP3 (T regulatory), and CD8 (T cytotoxic) using bladder tumor biopsy samples containing schistosome eggs, and found a Th2-skewed environment with elevated levels of FOXP3, suggesting that the downregulation of Th1 response through the action of FOXP3 on STAT4 may favor carcinogenesis (31). A meta-analysis for molecular markers related to schistosomal bladder cancer has also been performed (32).

Compared to clinical studies, studies using animal models allow for more experimental control; however, we face the challenge that a suitable animal infection model that completely recapitulates human infection does not exist. Specifically, mice or hamsters exposed to *S. haematobium* cercariae (the infective stage for humans) exhibit egg accumulation in the liver and intestines, rather than in the bladder, and while primate infections show similar pathophysiology to human infection, high costs, ethical concerns, and a dearth of species-specific reagents for the primate model collectively preclude extensive experimentation (14). To address this issue, our group developed a method to inject eggs directly into the bladder wall of mice, allowing us to replicate key pathological features of the human disease (17, 33). Here, we introduce recent studies that use this egg injection method and other methods to reveal important changes in host bladder biology related to *S. haematobium* infection, especially as it pertains to bladder carcinogenesis.

Mouse bladder wall injection with *S. haematobium* eggs leads to urothelial hyperplasia, a potentially pre-cancerous lesion (33). Microarray analysis comparing egg- and vehicle-injected bladders revealed decreased transcript levels of uroplakins in egg-injected organs, recapitulating other models of bladder damage that result in urothelial hyperplasia (18). The analysis also showed differential expression of genes belonging to cancer-associated and vascular endothelial growth factor (VEGF)-associated pathways, which is consistent with the observation that exposure to *S. mansoni* soluble egg antigen induces increased production of VEGF in human umbilical vein endothelial cells (34), as well as the report of elevated serum and urine VEGF protein levels in bladder cancer patients infected with *S. haematobium* (18, 35). These pathways provide an important starting point in further investigations to identify parasite and host molecules that give rise to schistosomal bladder cancer.

As with modulation of transcript expression, epigenetic changes in the host genome may also play a part in schistosomal bladder cancer. Inspired by the observation that DNA from the urine of patients infected with *S. haematobium* features hypermethylation of several genes correlated to degree of bladder damage (as evaluated by ultrasound) (36), our group conducted a similar study in egg-injected bladders and found a significant increase in DNA methylation in urothelial cells from parasite egg-injected bladders relative to vehicle-injected controls (19). Furthermore, drug-induced inhibition of DNA methylation resulted in decreased hyperplasia in the urothelium of the egg-injected bladders when compared to control animals that underwent egg-injection (19). These findings suggest a use for methylation-specific DNA sequencing as a potential method to detect schistosomal bladder cancer, although further study is necessary to help us identify methylation signatures specific for *S. haematobium* infection with bladder cancer (S+/C+), infection alone (S+/C-), cancer alone (S-/C+), uninfected individuals (S-/C-), as well as individuals with other sources of bladder damage (S-/C-/^*^).

Another “-omics” approach to deepen our understanding of schistosomal bladder carcinogenesis is proteomics. With a goal of identifying protein markers associated with schistosomal bladder cancer, Bernardo et al. characterized the urine proteomes from patients with or without *S. haematobium* infection and/or bladder cancer (S+/C+, S+/C–, S–/C+, S–/C–) and found different abundances of several proteins for each patient group (20). A similar study of the urine proteome used a larger patient pool but with less stringent inclusion criteria for bladder pathology (i.e., non-bladder cancer conditions were included), and it revealed, for each group, unique and overlapping host proteome profiles, as well as proteomic signatures characteristic of schistosomes (37).

Omics-relevant techniques to define the biology of schistosomal bladder cancer have also recently included microbiome experiments. A study of the urine microbiome from urogenital schistosomiasis patients showed differences in microbial communities between *S. haematobium*-infected and uninfected groups, as well as between advanced (infection with bladder pathology) and other groups (21). These findings demonstrate another possible method for the characterization and the diagnosis of schistosomal bladder cancer. Microbiome approaches could be combined with proteomics methods, allowing for the identification of bacterial proteins that may facilitate schistosomal bladder carcinogenesis.

Complementing “big data” studies, other work has focused on the specific host genes and pathways involved in the promotion of *S. haematobium*-associated bladder cancer. Chronic schistosome infection involves paired adult worms and their constant release of eggs. Both adult and egg stage parasites secrete molecules, inducing changes in the host microenvironment that may be pro-carcinogenic. Exposure to *S. haematobium* worm antigens increases proliferation and migration, and decreases apoptosis of Chinese hamster ovary (CHO) cells (22). Further experimentation demonstrated that the worm antigen-exposed CHO cells (but not the unexposed control cells) could generate tumors when injected subcutaneously into nude mice (23). The relevance of these findings, which are based on the use of ovarian rather than bladder-derived tissues, as well as a xenogeneic model, remains unclear. Moving to a more bladder-specific model, Botelho and coworkers introduced total adult antigen intravesically in mice, which produced urothelial dysplasia and inflammation (38). Inspection of the bladders with dysplasia revealed a mutation in the *KRAS* gene, a member of the *RAS* proto-oncogene family, suggesting a possible link between the occurrence of schistosomal bladder cancer and mutation in *KRAS* (24). We note that this study exposed the luminal side of the bladder urothelium to total worm antigen, whereas *S. haematobium*-infected hosts are exposed on the basal side of the bladder urothelium to egg antigens, as eggs are deposited in vesical blood vessels. Additionally, a single intravesical administration of the parasite extract is unlikely to recapitulate the location and degree of the host's exposure to parasite molecules found in a natural infection. We speculate that repeated intravenous administration of these products, perhaps intravenously, could produce more biologically relevant results.

Dysregulation of genes that control cell proliferation and death represents a core characteristic of cancer. In many cancers, dysfunction of the tumor suppressor gene *TP53* (and its protein product p53) allows the inappropriate survival of cells that would otherwise undergo apoptosis. Increased nuclear accumulation of p53 protein has been shown to correlate with mutations in the gene and with tumor grade in non-schistosomal bladder cancer (39), while in *S. haematobium-*associated bladder cancer, elevated p53 protein levels were found in both malignant and pre-malignant lesions, suggesting the potential use of p53 in the detection of schistosomal bladder cancer (40). Given the function of p53 as a tumor suppressor, we reasoned that its decreased expression would contribute to neoplastic changes in the *S. haematobium* egg-exposed bladder. We performed bladder wall egg injections on transgenic mice haploinsufficient for p53 in the bladder and found a lack of ulceration in the urothelium for p53-intact female mice compared to p53-haploinsufficient female mice and male mice (whether p53-sufficient or haploinsufficient). Since urothelial ulceration is a potential pathologic indicator of preneoplasia, these findings suggest the presence of host sex-specific factors that affect the pathogenesis of schistosomal bladder preneoplasia (25, 41). The study also served to establish a new method to examine the effect of disrupting a gene of interest in this animal model of *S. haematobium* infection. However, it remains unclear how to reconcile the findings of Santos et al. that p53 protein levels are increased in preneoplastic and neoplastic schistosomal bladder lesions (40) with our observation that p53-haploinsufficiency (which would result in decreased p53 protein levels) contributes to development of *S. haematobium* egg-induced, bladder neoplasia-associated lesions. While increased p53 levels in these lesions likely resulted from mutations in the p53 protein, determining whether the specific mutations leading to p53 accumulation directly contribute to neoplasia in schistosomal bladder lesions, e.g., ascertaining whether the mutation is a “passenger” vs. “driver” mutation [described in (42)], will require further investigation.

In another study using the mouse bladder wall egg injection method, Chala and others co-administered *S. haematobium* eggs and the cancer-inducing nitrosamine compounds N-nitrosodimethylamine (NDMA) and N-butyl-N-(4-hydroxybutyl) nitrosamine (BBN) (26). This work was inspired by a prior baboon *S. haematobium* infection model which demonstrated that sub-carcinogenic levels of nitrosamines become carcinogenic when combined with *S. haematobium* exposure (43). This complementation model lends credence to Knudson's multi-hit hypothesis as applied to schistosomal bladder carcinogenesis (44). The approach used by Chala et al. is also corroborated by the observation of elevated levels of nitrosamines in the urine of patients infected with *S. haematobium* compared to uninfected individuals (45), as well as by the recent finding that the BBN-induced mouse bladder cancer model and some human muscle-invasive bladder cancers feature similar mutation frequencies in key cancer-related genes (46). When Chala and co-authors combined nitrosamine exposure with mouse bladder wall egg injection, they noted abnormal histology and altered expression levels of cell adhesion genes *CDH1* (E-cadherin) and *VIM* (vimentin), the proliferation marker *MKI67* (Ki-67), and *TP53* (p53) (26). Interpretation of findings from this study is potentially confounded by several considerations: (1) presumably unblinded histological analyses, (2) use of a suboptimal egg dose injection (acknowledged by the authors), (3) use of eggs from frozen urine samples (e.g, dead eggs unable to secrete proteins), (4) lack of a time course sufficiently long enough to observe frankly neoplastic lesions, and (5) use of a high BBN concentration that, in our hands, results in a potent BBN-mediated procarcinogenic effect that may mask any procarcinogenic influence of *S. haematobium* eggs. Chala and co-authors are to be applauded for their efforts, but deeper studies will be mandatory to better elucidate the pathophysiology of schistosomal bladder carcinogenesis.

In the mouse bladder wall injection model, the preneoplastic changes and alteration in the described genes and pathways arise as a response to the damage associated with the retention and/or traversal of the eggs across the urothelium, as well as to the immunomodulatory egg-secreted molecules. Importantly, we note that the model delivers one installment of parasite eggs, giving only an acute view of their effect, while in contrast, infection with the adult parasites results in the continuous deposition of eggs. In turn, this chronic egg-induced bladder damage leads to increased inflammation and cell turnover, eventually giving rise to carcinoma. Improvement of the mouse model to facilitate chronic infection with egg-laying adult *S. haematobium* worms would allow for further investigation and identification of host and parasite molecules, genes, and pathways uniquely affected in schistosomal bladder cancer. Key comparisons would include parasite-infected mice with bladder cancer (S+/C+) and mice with only bladder cancer (S-/C+), as studied in patients (29, 47).

## Identification of key parasite molecules

The mouse bladder wall egg-injection studies discussed above have focused on changes in the biology of the host in response to the presence of *S. haematobium* eggs. Given that these host-side changes arise from a host-parasite interaction, a related line of investigation follows: determination of the identity of specific schistosome molecules that induce the host-side changes. Very few studies have focused on the parasite side of the host-parasite interaction, likely owing to the difficulty of isolating and analyzing parasite components. In contrast to the studies on host-side changes, schistosome molecular studies have mainly used *in vitro* methods, which, because of the remoteness from the original biological context, come with the caveat that the results may have limited relevance, but allow us to finely probe the function of such molecules and their effects on host cells.

Parasite-derived estrogen-related compounds and metabolites, found during a study of hypogonadism in schistosomiasis patients (48), have been proposed to play a role in the development of schistosomal bladder cancer. Proteomic analysis of human urine containing *S. haematobium* eggs revealed the presence of several products not found in urine from uninfected individuals (49), including estrogen-like metabolites and guanine-derived oxidation products, which can both give rise to genetic mutation and carcinogenesis (27, 50). Estrogen-like metabolites were also found in *S. haematobium* soluble egg antigen (SEA), which, when administered to urothelial cells, induced key cancer phenotypes, including increased cell proliferation and decreased apoptosis (28). These findings suggest that parasite-derived estrogen-related compounds may contribute to the development of schistosomal bladder cancer and that they may be useful as markers for schistosome infection, if not for schistosomal bladder cancer (27, 51). However, the relationship between these estrogen-related compounds and the observation that bladder cancer is more common in men (who have higher androgen:estrogen ratios) remains to be reconciled.

Like the eggs of other schistosome species, *S. haematobium* eggs secrete a variety of proteins, which modulate the host immune response and may facilitate egg escape from the host. Among the most abundant of these proteins is H-IPSE, the *S. haematobium* ortholog to *S. mansoni* IL-4 inducing principle of *S. mansoni* eggs (IPSE/alpha-1). Our studies have identified many interesting features of H-IPSE, including its ability to infiltrate host cells and translocate to the nucleus (52). Akin to the *S. mansoni* ortholog, H-IPSE can induce IL-4 production, bind immunoglobulins, cytokines, and chemokines, skew cell cycle state toward the S phase, and increase cell proliferation (unpublished observations). During chronic infection with *S. haematobium*, the continuous deposition of eggs in the bladder and subsequent retention and traversal of the eggs through the urothelium likely results in damage to the tissue. At the same time, however, H-IPSE released from the eggs may help to counteract the damage to the urothelium by increasing cell proliferation. We speculate that this increased cell proliferation in the urothelium contributes to malignancy associated with *S. haematobium* infection.

These studies represent some promising initial steps in identifying links between parasite molecules and host genes that contribute to bladder cancer. Further investigations could test the carcinogenic potential of other schistosome molecules, starting with those found in proteomic profiling studies of eggs and adults. Additionally, administration of such molecules in an adaptation of the current mouse bladder wall injection model of *S. haematobium* infection would help corroborate the findings of *in vitro* studies.

## Possible mechanisms of schistosomal bladder cancer

We generally accept the idea that urogenital schistosomiasis contributes to, but alone is probably insufficient for oncogenesis. The concurrent presence of additional carcinogens, including environmental exposures (intake of nitrosamine compounds through smoking or diet), genetic predisposition (loss-of-function mutation in tumor suppressor genes or gain-of-function mutation in oncogenes), and other infections (uropathogenic *E. coli*, HPV), could help accelerate the development of schistosomal bladder cancer (14). However, the understanding of the specific components of *S. haematobium* infection that lead to bladder cancer is in its infancy. Here, we discuss possible mechanisms through which urogenital schistosomiasis can give rise to bladder cancer.

*S. haematobium* infection leads to egg deposition in the bladder wall, with some eggs presumably causing damage by their traversal through the urothelium to the bladder lumen. The remaining eggs (that fail to traverse) cause damage through chronic inflammation by their interaction with host immune cells and subsequent granuloma formation (53). The bladder urothelium consists of multiple layers of different cell types, which, in response to injury, dramatically increase their otherwise slow turnover rate [reviewed in (54)]. Combining these observations for *S. haematobium* infection and urothelial regeneration leads to the hypothesis that infection-induced damage and chronic inflammation in the urothelium contribute heavily to the development of bladder cancer (55, 56). Indeed, the idea that sites of inflammation can give rise to cancer has been proposed in the mid-1800s by Virchow [reviewed in (57)], and, more recently, dysbiosis in the microbiome has also been suggested as a risk factor (58, 59). While current studies on the urine microbiome of patients (with different binary combinations of schistosome infection and bladder pathology) have identified the abundance and distribution of microbiota species in each case, future studies could delve into detailed associations by introducing more parameters, such as the degree of bladder pathology and parasite infection burden.

Upregulation of cell proliferation and injury repair mechanisms in response to egg traversal-associated urothelial damage also likely contributes to the development of schistosomal bladder cancer. The concurrent inflammation and presence of possibly mutagenic parasite molecules could explain the observation of increased chromosomal damage found in urogenital schistosomiasis patients (55). As discussed earlier, estrogen-like metabolites of schistosome origin may also drive an increase in the rate of gene mutation by forming adducts with DNA (50). Accumulation of these potentially mutagenic events over many cell generations could eventually give rise to dysfunctional or even cancerous cells.

Aside from the response to physical damage, the increase in urothelial cell proliferation can result from the action of *S. haematobium* egg secretions. Soluble egg antigen and IPSE, a highly abundant constituent of soluble egg antigen, have both shown the ability to increase cell proliferation in endothelial cells, corroborating the association of angiogenesis with egg granuloma formation, and reinforcing the idea that angiogenesis may be another link between schistosome infection and bladder cancer (60). The treatment of human umbilical vein endothelial cells with *S. mansoni* soluble egg antigen resulted in upregulation of VEGF (34), consistent with the observation of perturbation in the VEGF pathway following mouse bladder wall injection of *S. haematobium* eggs, as well as the observation of increased vascularization in the genital mucosa of women infected with *S. haematobium* compared to uninfected individuals (61). Given that IPSE increases cell proliferation, upcoming studies identifying its host gene targets may find that it modulates VEGF expression and genes in other pathways, such as Hedgehog (Hh) and Wingless (Wnt) pathways, which also play a role in cell proliferation (54).

## Future directions in schistosomal bladder cancer research

The introduction of the mouse bladder wall injection model has helped to characterize the pathologic, and possibly carcinogenic, changes in the host bladder in the presence of *S. haematobium* eggs. The ability to recapitulate natural bladder oviposition in the mouse represents the next challenge in improving the mouse model and would allow us to avoid artifacts associated with the egg injection procedure, as well as observe the effect of chronic oviposition in contrast to one-time egg administration. Similarly, with the identification of potentially carcinogenic parasite molecules, such as the estrogen-like metabolites and IPSE, methods for chronic local *in vivo* administration of these factors would help to strengthen the evidence for their carcinogenicity. More ambitiously, the development of a method to generate transgenic schistosomes that carry stable genomic modifications, such as gene disruptions and reporter gene insertions, would further the study of not only carcinogenic schistosome molecules but also schistosome genes and their role in the development of the parasite within the host, eventually leading to the identification of novel drug targets and therapeutics for the prevention and treatment of schistosomiasis.

## Author contributions

All authors listed have made a substantial, direct and intellectual contribution to the work, and approved it for publication.

### Conflict of interest statement

The authors declare that the research was conducted in the absence of any commercial or financial relationships that could be construed as a potential conflict of interest.
